# Discovery of novel solid solution Ca_3_Si_3−*x*_O_3+*x*_N_4−2*x*_: Eu^2+^ phosphors: structural evolution and photoluminescence tuning

**DOI:** 10.1038/s41598-017-18319-5

**Published:** 2017-12-22

**Authors:** Baochen Wang, Yan-gai Liu, Zhaohui Huang, Minghao Fang, Xiaowen Wu

**Affiliations:** Beijing Key Laboratory of Materials Utilization of Nonmetallic Minerals and Solid Wastes, National Laboratory of Mineral Materials, School of Materials Science and Technology, China University of Geosciences, Beijing, 100083 China

## Abstract

Discovery of novel phosphors is one of the main issues for improving the color rendering index (CRI) and correlated color temperature (CCT) of white light-emitting diodes (w-LEDs). This study mainly presents a systematic research on the synthesis, crystal structure variation and photoluminescence tuning of novel (oxy)nitride solid solution Ca_3_Si_3−*x*_O_3+*x*_N_4−2*x*_: Eu^2+^ phosphors. XRD refinements show that lattice distortion occurs when *x* value diverges the optimum one (*x* = 1). The lattice distortion causes a widening of emission spectrum and an increase of Stokes shift (ΔSS), which leads to a bigger thermal quenching. With decrease of *x* value, the emission spectrum shows an obvious red-shift from 505.2 to 540.8 nm, which is attributed to the crystal field splitting. The enhanced crystal field splitting also broadens the excitation spectrum, making it possible to serve as the phosphor for near ultraviolet (n-UV) LEDs. A 3-phosphor-conversion w-LED lamp was fabricated with the as-prepared phosphor, which exhibits high CRI (Ra = 85.29) and suitable CCT (4903.35 K). All these results indicate that the Ca_3_Si_3−*x*_O_3+*x*_N_4−2*x*_: Eu^2+^ phosphor can serve as the green phosphor for n-UV w-LEDs, with a tunable spectrum by controlling the crystal structure and morphology.

## Introduction

In recent decades, w-LEDs are marching on the ultimate goal to replace all incandescent bulbs and compact fluorescent lamps to provide an energy efficient and long-lasting option for general illumination^1–3^. Compared with conventional lighting lamps, w-LEDs have overwhelming superiorities including longer lifetimes, lower energy consumption, and an environmentally friendly design without the need for mercury^4,5^. Though w-LEDs have developed rapidly since the encapsulation of the first and most common YAG: Ce^3+^ (Y_3_Al_5_O_12_: Ce^3+^) converted w-LED, further progress should be made in terms of CRI and CCT of w-LEDs for general lighting^5,6^.

Phosphors are commonly used to generate a broad spectrum of white light in w-LEDs. The three most popular approaches are a blue LED with a yellow phosphor; an ultraviolet LED with blue and yellow phosphors (or red, green and blue phosphors); and a device that combines red, green and blue LED chips^7,8^. Unfortunately, each of the schemes suffers some drawbacks. The disadvantage of using a blue LED and a yellow phosphor is low CRI (<80) and high CCT (>4000 K), which are undesirable for indoor use^9^. The second strategy can provide high CRI value, but at the expense of poor efficacy^5^, while the third approach may generally be the most expensive option^1^. In short, efforts are required to address issues of higher efficiency, better color rendition and smaller thermal quenching in terms of phosphors for w-LEDs. Discovery of novel phosphors and optimization of strategies for realizing white light will be two dominating approaches to developing consummate w-LEDs.

Combinatorial chemistry, single-particle diagnosis and solid solution are three main approaches to discovering new phosphors^7^. Among them, solid solution is the easiest and most cost-efficient way, especially for nitride, because structural diversity and different O/N ratio of (oxy) nitride greatly influence the 5d energy level of rare earth ions, which contributes to tunability of luminescence spectrum^10,11^. Based on these three strategies, a variety of phosphors have been developed, including oxides, oxyfluorides, sulfide, phosphate and (oxy) nitrides^12^. (Oxy)nitride phosphors have received significant attention in recent years due to their encouraging luminescent properties, such as excitability by blue light, high conversion efficiency, the possibility of full color emission, as well as their low thermal quenching and high chemical stability. All these merits make (oxy)nitride phosphors potential for use in w-LEDs^13^. The well-studied (oxy)nitride phosphors include α-sialon^11,14–17^, β-sialon^18,19^, Sr_2_SiN_*z*_O_4–1.5*z*_: Eu^2+^ ^20^, Sr_2−*y*−*z*_Ca_*z*_Si(O_1−*x*_N_*x*_)4: *y*Eu^2+^ ^21^, Sr_1−*x*−*y*−*z*_Ca_*x*_Ba_*y*_Si_2_O_2_N_2_:*z*Eu^2+^ ^22^, MSi_2_O_2−*δ*_N_2+2/3*δ*_ (M = Ca, Sr, Ba)^23^, Ba_4_Si_6_O_16–3*x*/2_N_*x*_^24^ and so on.

Ca_3_Si_2_O_4_N_2_ is a new phase found in recent decades. The X-ray powder diffraction (XRD) data of Ca_3_Si_2_O_4_N_2_ were first given by ZK Huang *et al*.^25^. After that, Ali Sharafat *et al*.^26^ reported the refine crystal structure of an analogue Ca_2.89_Si_2_N_1.76_O_4.24_ with cubic lattice. Later, XM Wang *et al*.^27^ discussed the O/N ordering and discovered that this interesting phase is composed of corrugated 12-membered ring structure. Although several reports on photoluminescence properties of Eu^2+^ or Ce^3+^ doped Ca_3_Si_2_O_4_N_2_ have been published in recent years, research on this potential host as a phosphor is still at a primary stage^28–30^.

This study mainly aims at the preparation, characterization of phase composition and crystal structure as well as luminescence properties of the novel (oxy) nitride solid solution Ca_3_Si_3−*x*_O_3+*x*_N_4−2*x*_: Eu^2+^ phosphor. The mechanism how structural evolution affects the luminescence spectra of title phosphor will be the main issue in this paper. The results present that the increasing N/O ratio and structural distortion significantly affect the luminescent properties, such as crystal field splitting, stokes shift, peak shifts, thermal stability and so on.

## Experimental

### Raw materials and synthesis

The Ca_3_Si_3−*x*_O_3+*x*_N_4−2*x*_: Eu^2+^: 0.03Eu^2+^ (*x* = 0, 0.2, 0.3, 0.4, 0.5, 0.6, 0.8, 0.9, 1, 1.2, 1.4 and 1.5) phosphors were synthesized by conventional high-temperature solid-state reaction method using starting materials CaCO_3_ (AR, Westlong Share Ltd., Guangdong, China), SiO_2_ (AR, Sinopharm Group Chemical Reagent Ltd., Shanghai, China), α-Si_3_N_4_ (3N, Aladdin Share Ltd., Shanghai, China) and Eu_2_O_3_ (4 N, Minmetals Rare Earth Ltd., Beijing, China). The stoichiometry amount of raw materials was thoroughly grounded in an agate mortar. Then the mixtures were put into an alumina crucible and sintered at 1430 °C for 4 h in a flowing 10%H_2_/90%N_2_ atmosphere. Finally, the samples were furnace-cooled to room temperature. Afterwards, the as-prepared samples were ground into powders for further measurements.

### Characterizations

The phase composition of as-synthesized samples was identified by x-ray diffraction (XRD; D8 FOCUS diffractometer, Germany) with graphite-monochromatized Cu Kα radiation (λ = 1.5406 Å). Photoluminescence emission (PL) and photoluminescence excitation (PLE) spectra were measured by F-4600 fluorescence spectrophotometer (Hitachi, Japan) with a photomultiplier tube functioning at 500 V, and a 150 W Xe lamp as the excitation source. The spectral resolution for photoluminescence measurements was 1 nm. Temperature dependent luminescence properties were performed on the same spectrophotometer equipped with an automatic temperature regulating device. Diffuse reflection spectra were obtained via a UV-3600 UV-Vis-NIR spectrophotometer (Shimadzu) connected with an integrating sphere. The Debye temperatures and Young’s modulus were calculated by first principle in the Cambridge Sequential Total Energy Package (CASTEP). Throughout the computational process, ultrasoft pseudopotential, Perdew-Burke-Ernzerhof generalized gradient approximation (GGA-PBE), 340 eV plane-wave energy cutoff and 2 × 2 × 2 Monkhorst-Pack grids were used. The geometry optimization was performed by using the Damped molecular dynamics (Damped MD) algorithm. The convergence tolerance of energy was 1 × 10^−5^ eV/atom.

## Results and Discussion

### Phase and crystal structure

**Figure 1:**
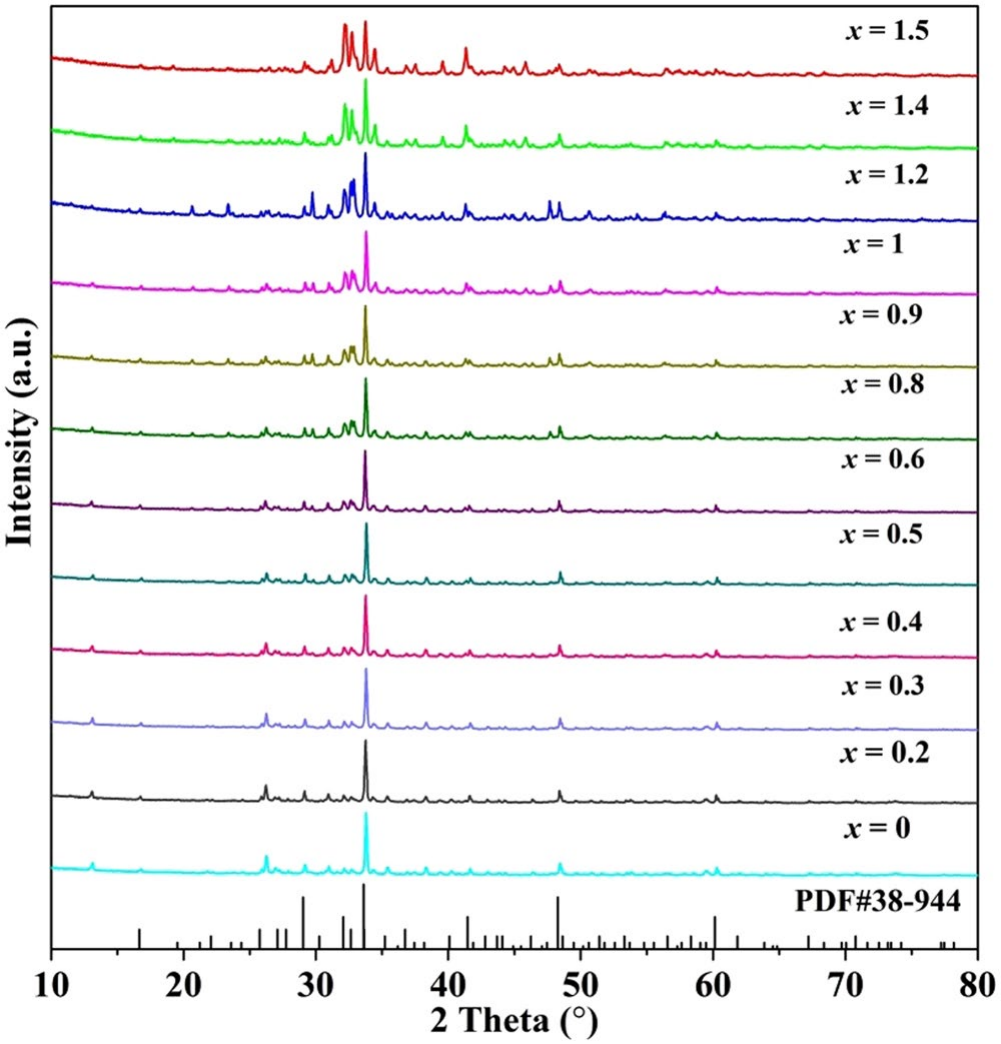
XRD patterns of Ca_2.97_Si_3−*x*_O_3+*x*_N_4−2*x*_: 0.03Eu^2+^ samples and the standard pattern (PDF 38-944) of Ca_3_Si_2_O_4_N_2_.

The phase composition of as-synthesized phosphors was identified by XRD. The XRD patterns of all Ca_2.97_Si_3−*x*_O_3+*x*_N_4−2*x*_: 0.03Eu^2+^ samples and the standard pattern of Ca_3_Si_2_O_4_N_2_ (PDF 38–944)^31^ are provided in Fig. 1. The diffraction peaks matched well with the standard pattern, demonstrating that introduction of Eu^2+^ ions doesn’t change the phase, nor cause any impurities. This result indicates the formation of a series of solid solution phosphors in the range of *x* values from 0 to 1.5. In addition, it can be seen that the intensity of the main diffraction peak around 33 degree ((440) crystal plane) declines obviously with increase in *x*. After excluding external effects, this change is considered to result from the variation of atom quantity and distribution in crystal. These effects can be described by structure factor (*F*_*hkl*_). The relationship between peak intensity and *F*_*hkl*_ is described in equation (1)^32^:

1 $${I}_{hkl}\propto {|{F}_{hkl}|}^{2}$$

With decrease of *x* value, N atom and the total atom quantity in each unit cell increase, which may induce a preferential crystal growth along (440) plane. Hence, the amplitude of the X-ray composite wave scattered by the atoms in (440) direction enhanced and *F*_*hkl*_ along (440) direction becomes larger. As described in equation (1), enhancement of *F*_*hkl*_ will certainly lead to the intensity increase of (440) peak.

**Figure 2:**
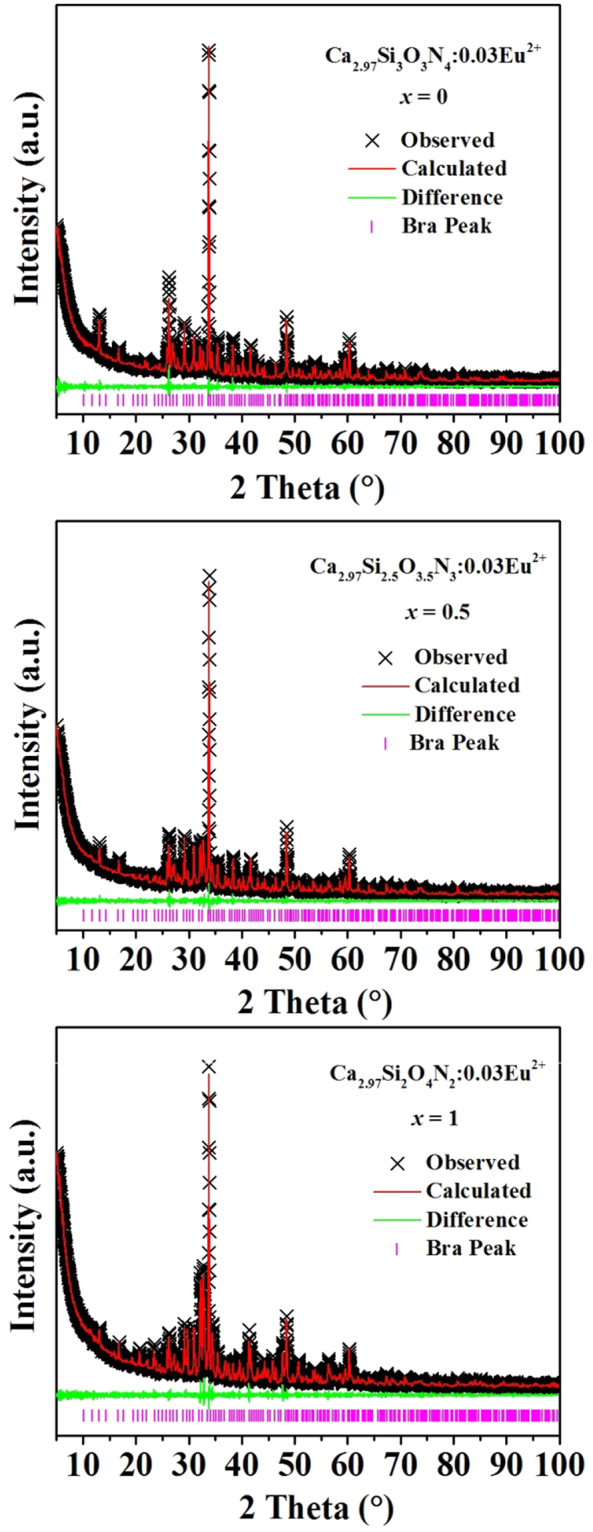
XRD refinement patterns of Ca_2.97_Si_3−*x*_O_3+*x*_N_4−2*x*_:0.03Eu^2+^ (*x* = 0 (**a**), 0.5 (**b**) and 1 (**c**)) phosphors. Black Crosses represent the observed diffraction peak; red lines indicate the refined patterns; green lines are residuals and purple tick marks show the Bragg positions.

To further determine the phase purity and crystal structure, the XRD patterns of three representative samples (*x* = 0, 0.5 and 1) were refined by the Rietveld refinement method using the Topas program. The standard pattern of Ca_3_Si_2_O_4_N_2_ (PDF 38-944; a = b = c = 15.07 Ǻ; V = 4325.10 Ǻ^3^) is referenced as an initial structural model. The refinement patterns are illustrated in Fig. 2 and the main refinement parameters and detailed crystallographic data are given in Table 1. All structure refinements are convergent and end with acceptable and publishable *R* factors. The results of the refinement further demonstrate that these phosphors match well with the starting model (Ca_3_Si_2_O_4_N_2_) and the series of solid solution phosphors are single phase without any impurities or secondary phases.

**Table 1:** The main refinement parameters and detailed Crystallographic data of Ca_2.97_Si_3−*x*_O_3+*x*_N_4−2*x*_:0.03Eu^2+^ (*x* = 0, 0.5 and 1).

Compound	*x* = 0	*x* = 0.5	*x* = 1
Space Group	*P*a $\bar{3}$	*P*a$\bar{3}$	*P*a$\bar{3}$
*a* (Å)	15.0749(7)	15.0699(8)	15.0788(13)
*V* (Å^3^)	3425.84(51)	3422.42(55)	3433.32(103)
*α* (°)	90	90	90
2*θ*-interval (°)	5–100	5–100	5–100
*R*_wp_ (%)	5.339	4.982	4.975
*R*_exp_ (%)	3.433	3.484	3.369
*R*_B_ (%)	0.478	0.418	0.259
GOF	1.555	1.430	1.477

**Figure 3:**
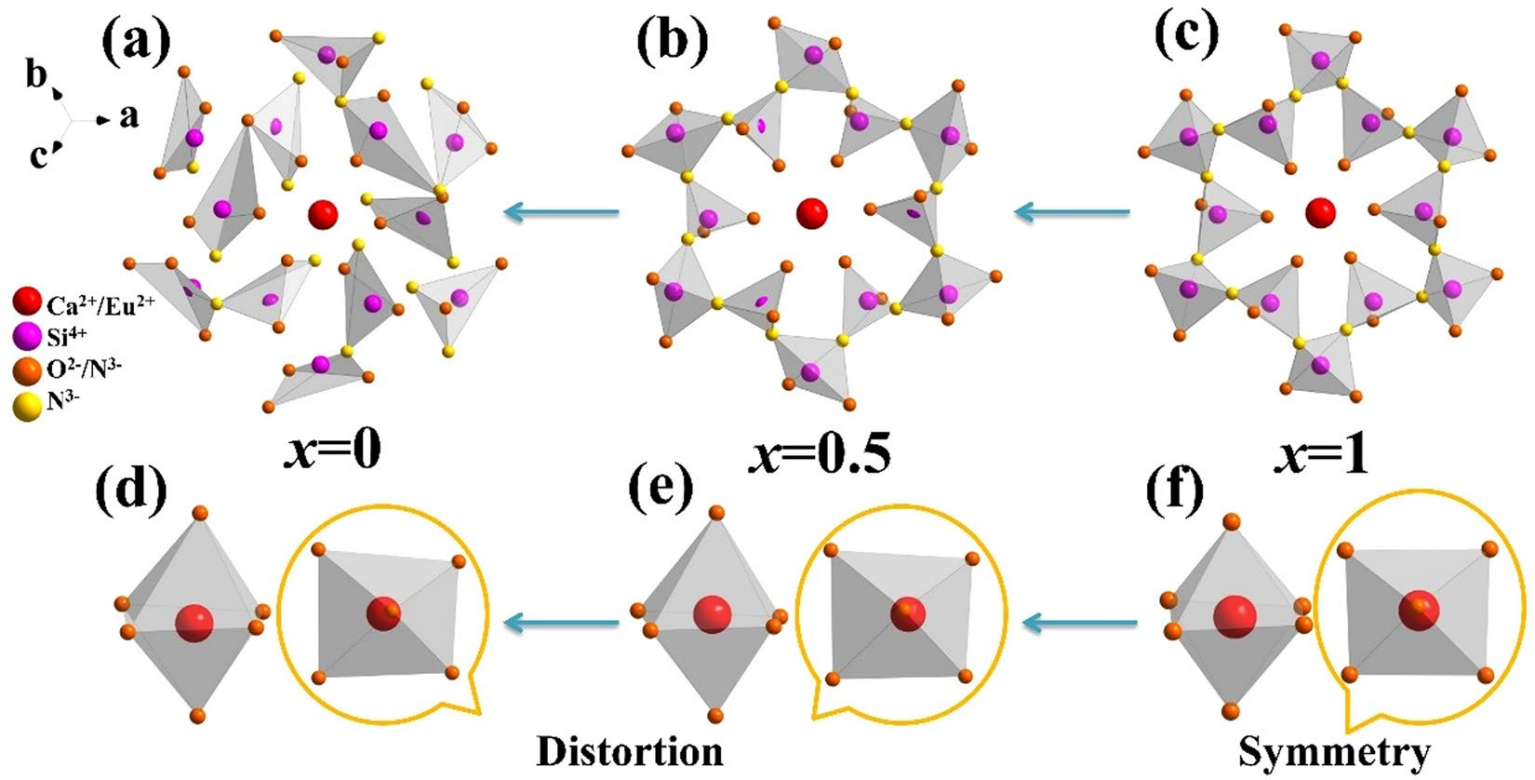
Schematic structure evolution of Ca_2.97_Si_3−*x*_O_3+*x*_N_4−2*x*_: 0.03Eu^2+^ phosphors depending on *x* values (*x* = 0, 0.5 and 1, respectively). (**a**–**c**) Represent the distortion of corrugated 12-membered ring structure and (**d**–**f**) show the distorted coordination environment of Ca4 site with decreasing *x* values.

To show the structure evolution of Ca_2.97_Si_3−*x*_O_3+*x*_N_4−2*x*_: 0.03Eu^2+^ phosphors, the schematic crystal structures depending on XRD refinements are shown in Fig. 3. As reported by Wang *at al*.^27^, Ca_3_Si_2_O_4_N_2_ is composed of 12-membered rings of [SiO_2_N_2_] tetrahedra. Similar12-membered rings are also found in the three refined structures. It can be found in Fig. 3(c) that the 12-membered rings (for *x* = 1) are highly symmetric, while a slightly distorted one (for *x* = 0.5) is displayed in Fig. 3(b) due to substitution and impaction of N ions. When *x* reaches 0, a serious distortion is observed and the 12-membered rings are severely damaged, as shown in Fig. 3(a). Although the 12 distorted tetrahedra still hold a basic shape of 12-membered rings, some of the tetrahedra are disconnected. This variation may probably be caused by introduction of more N ions. To show the evolution of the coordination environment of central activator ions, the coordination polyhedra of Ca4 site are illustrated in Fig. 3(d–f). The Ca4 site shows an octahedral coordination environment. Viewing perpendicularly to the octahedral plane, one can see that the coordination octahedron is highly symmetric when *x* = 1, while the symmetry of the octahedron decreases with reduction of *x* due to lattice distortion. It’s worth noting that although there are seven different car sites in the whole structure^27^, distortion is believed to occur on all the Ca sites. Structure evolution mentioned above would have an important influence on the luminescence properties of the title phosphor.

**Figure 4:**
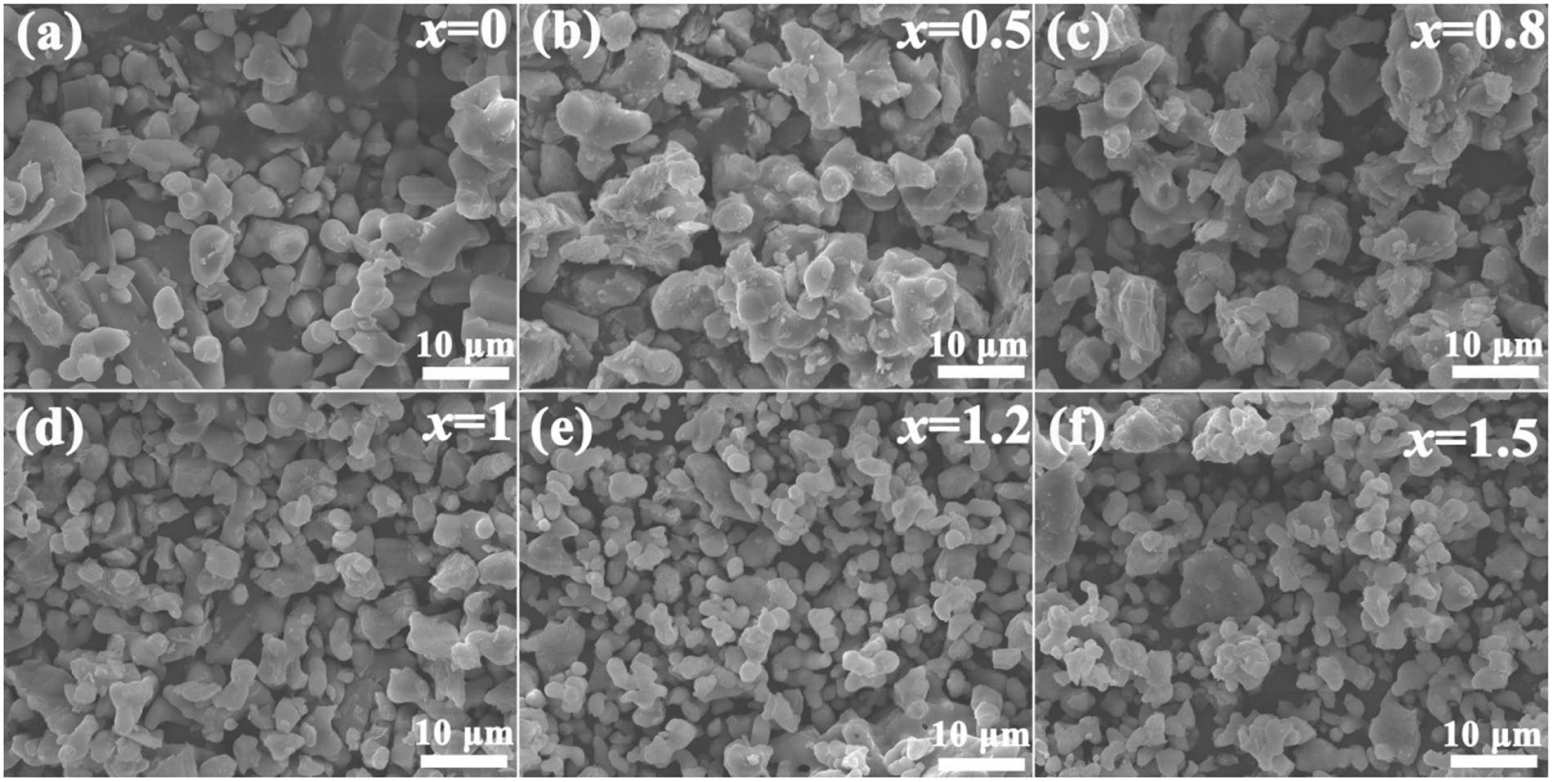
Scanning electron micrographs of Ca_2.97_Si_3−*x*_O_3+*x*_N_4−2*x*_: 0.03Eu^2+^ (*x* = 0 (**a**), 0.5 (**b**), 0.8 (**c**), 1 (**d**), 1.2 (**e**) and 1.5 (**f**)) phosphors.

The particle size and distribution of powders may influence the luminescence properties of phosphors^33^. Accordingly, the surface microtopography of selected samples (*x* = 0, 0.5, 0.8, 1, 1.2 and 1.5) was observed by SEM and the corresponding images are illustrated in Fig. 4. It can be obviously identified that all the samples have a homogeneous morphology and the particle sizes diminish with the increasing *x* values. At lower *x* values shown in Fig. 4(a–c), some of the primary particles reunited to yield agglomerates, and the observed “sintering necks” are suggesting that agglomeration has occurred during the synthesis procedure. By contrast, at higher *x* values shown in Fig. 4(d–f), the particles are well decentralized with smaller sizes. These phenomena indicate that the degree of crystal growth is well promoted in the nitrogen-rich case with lower *x* values.

**Figure 5:**
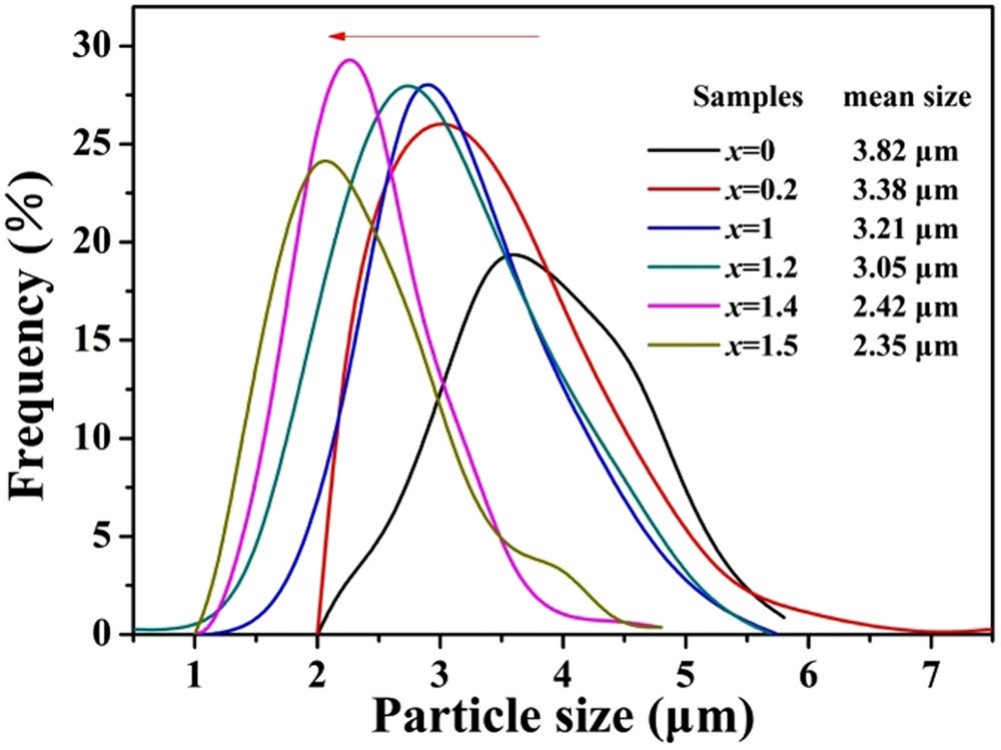
Granulometric distribution of selected Ca_2.97_Si_3−*x*_O_3+*x*_N_4−2*x*_: 0.03Eu^2+^ phosphors (*x* = 0, 0.2, 1, 1.2, 1.4 and 1.5).

To better understand the evolution of particle sizes with solid solubility, the granulometric distribution of selected samples (*x* = 0, 0.2, 1, 1.2, 1.4 and 1.5) were counted according to the SEM images and the distribution curves are illustrated in Fig. 5. It’s obvious that the center of distribution curves shifts to smaller values with increasing *x*. The mean particle sizes are in the range from 3.82 for *x* = 0 to 2.35 μm for *x* = 1.5. The particle size reduced approximately by half and the statistical results are in agreement with the macroscopic SEM images in Fig. 4. The emission spectra of phosphors are a sum of emissions from luminescence center at the surface and in the interior of the particles. As the particles grow smaller, the concentration of surface activator ions steadily increases. With the crystallite enlargement, more and more activator ions are located in the interior of the crystals, rather than at the near surface where energy can rapidly transfer to surface defects and then be consumed by high vibrational energies^34^. That is to say, the enlargement of particles can effectively suppress energy transfer to the crystal surface to result in efficient luminescence by confining the ions in the interior core of the grains^35^.

**Figure 6:**
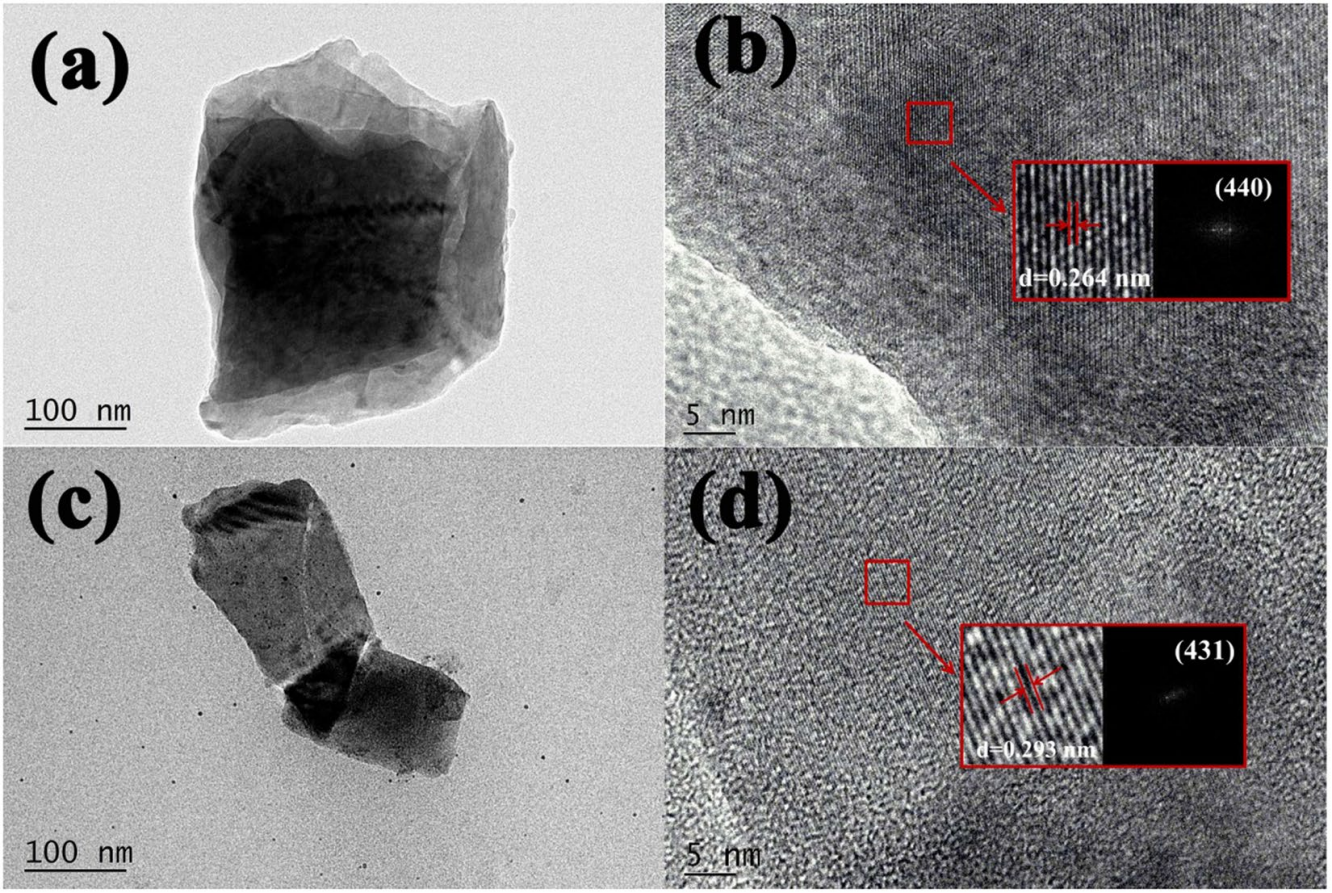
(**a**) TEM image, (**b**) HRTEM images of Ca_2.7_Si_2.8_O_3.2_N_3.6_: 0.03Eu^2+^ (*x* = 0.2); (**c**) TEM image, (**d**) HRTEM images of Ca_2.7_Si_1.5_O_4.5_N: 0.03Eu^2+^ (*x* = 1.5); inset in (**b** and **d**) shows the FFTs of the corresponding HRTEM images.

The fine local structures of Ca_2.97_Si_3-*x*_O_3+*x*_N_4−2*x*_: 0.03Eu^2+^ phosphors (*x* = 0.2 and 1.5) were further verified by HRTEM and fast Fourier transform (FFT) techniques. The relevant images are presented in Fig. 6. The lattice fringes in Fig. 6(b,d) with d spacing of 0.2635 and 0.2930 nm could be assigned to the (440) and (431) plane of crystal Ca_2.97_Si_3-*x*_O_3+*x*_N_4−2*x*_: 0.03Eu^2+^. The conventional d spacing of corresponding planes is 0.2665 and 0.2952 nm for Ca_3_Si_2_O_4_N_2_ (PDF 38-944), respectively. The d spacing of the principal planes (440) of Fig. 6(d) was calculated to be 0.2641 nm according to the Bragg equation^36^. The lattice fringes have changed slightly owing to cell volume increase. This result is consistent with the structural refinement where the cell volumes increase from 15.0706 to 15.0788 Å^3^. The continuous lattice fringes further confirm the high crystalline nature of full series of the solid solutions.

**Figure 7:**
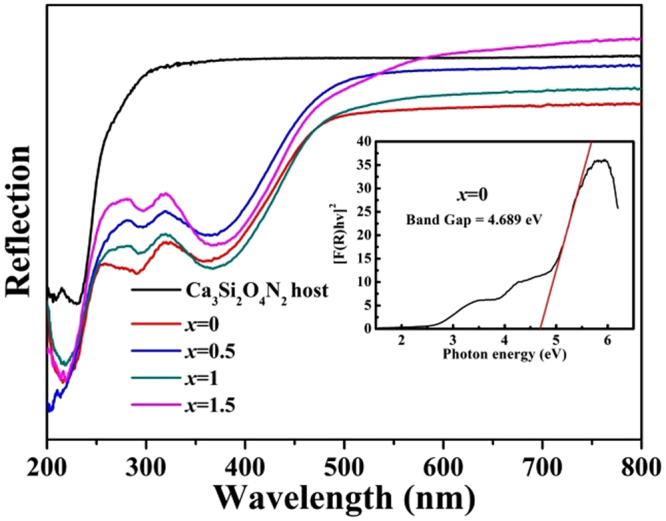
DRS of Ca_3_Si_2_O_4_N_2_ host and Ca_2.97_Si_3−*x*_O_3+*x*_N_4−2*x*_: 0.03Eu^2+^ phosphors (*x* = 0, 0.5, 1 and 1.5). The inset shows the absorption spectrum of Ca_2.97_Si_3_O_3_N_4_: 0.03Eu^2+^ host calculated using the Kubelka-Munk equation.

For analysis of absorption spectroscopy, diffuse reflection spectrum (DRS) of Ca_3_Si_2_O_4_N_2_ host and typical samples (*x* = 0, 0.5, 1 and 1.5) are measured and shown in Fig. 7. The Ca_3_Si_2_O_4_N_2_ host has one energy absorption band with the maximum absorption at 230 nm. By contrast, the Eu^2+^ doped samples have three absorption bands centering at 220, 297 and 360 nm, respectively. Obviously, the absorption at 220 nm is assigned to the host lattice absorption and the last two bands are attributed to the energy level transitions. To further discuss the energy levels, the energy gap was calculated according to the Kubelka-Munk function:

2 $${[{F}({{R}}_{\infty }){hv}]}^{{\rm{n}}}={C}({hv}-{{E}}_{{g}})$$

The band gap of Ca_3_Si_2_O_4_N_2_ host was calculated to be approximately 4.689 eV based on above equation^37^.

**Figure 8:**
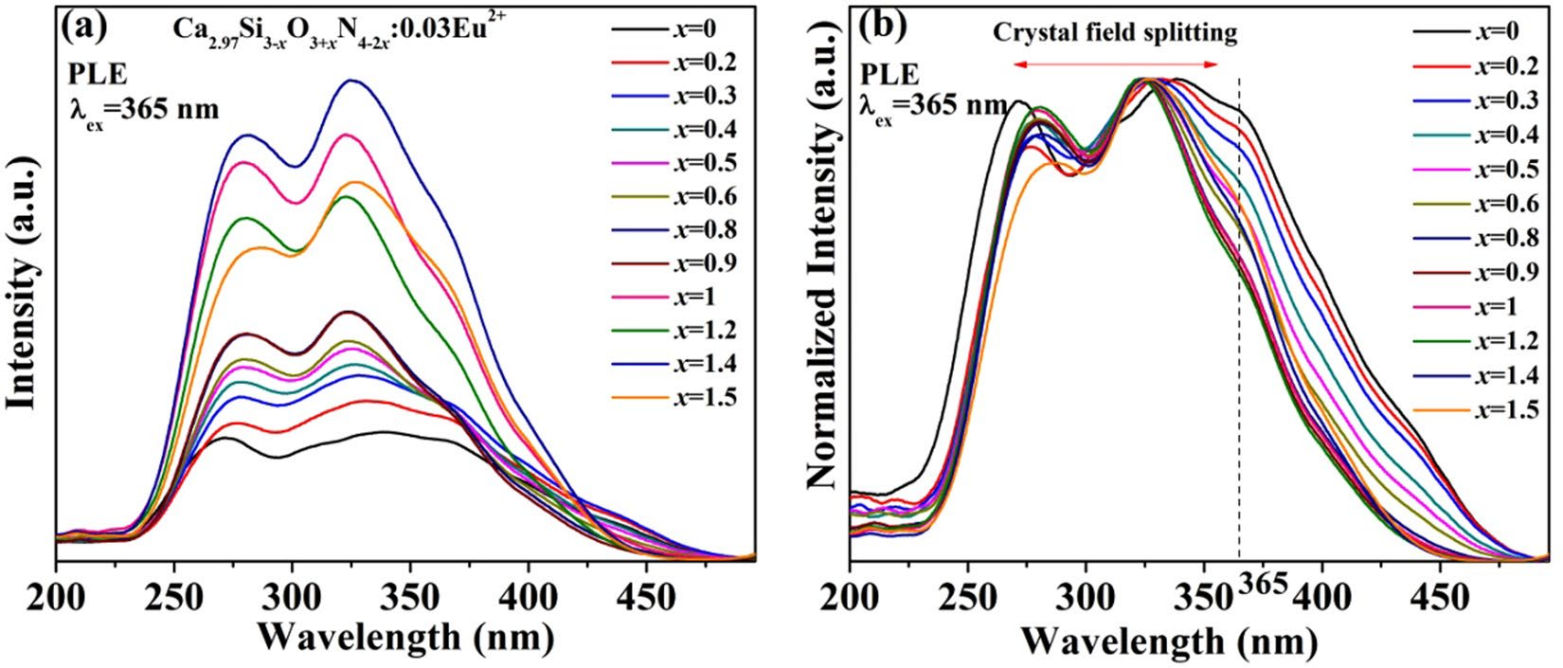
(**a**) PLE and (**b**) normalized PLE spectra of Ca_2.97_Si_3−*x*_O_3+*x*_N_4−2*x*_: 0.03Eu^2+^ phosphors.

The photoluminescence excitation (PLE) spectra of all Ca_2.97_Si_3−*x*_O_3+*x*_N_4−2*x*_: 0.03Eu^2+^ phosphors are illustrated in Fig. 8. The PLE spectra have three peaks at 270, 340 and 365 nm attributed to 4f^7^→4f^6^5d^1^ transition. In the normalized PLE spectra shown in Fig. 8(b), the excitation peaks at 270 nm show a blue-shift while the other two peaks present a red-shift with decreasing *x* values. Hence, the entire excitation bands are broadened due to the crystal field splitting effect^34^. When the *x* values decrease, the nitrogen-rich phosphors are obtained and Eu^2+^ ions have opportunities to coordinate with more nitrogen atoms. High formal charge of N^3−^ causes bigger molecular orbital overlap and increases the degree of covalency, which increases the centroid shift and crystal field splitting of Eu^2+^ ions^38^. As reported by Yi-Chen Chiu, *et al*.^28^, the Ca_3_Si_2_O_4_N_2_: Eu^2+^ phosphor has a maximum excitation wavelength at 328 nm and emits 510 nm green lights. It cannot be effectively excited by n-UV light (365–380 nm) and the emission isn’t pure green. These properties indicate that it is not suitable for n-UV w-LEDs. However, by modulation of the crystal structure, the excitation band was successfully broadened and the Ca_3_Si_3_O_3_N_4_: 0.03Eu^2+^ (*x* = 0) phosphor can be effectively excited by n-UV lights ranging from 365 to 375 nm. This tuning of photoluminescence is important for realizing the practicability of the phosphors for n-UV w-LEDs.

**Figure 9:**
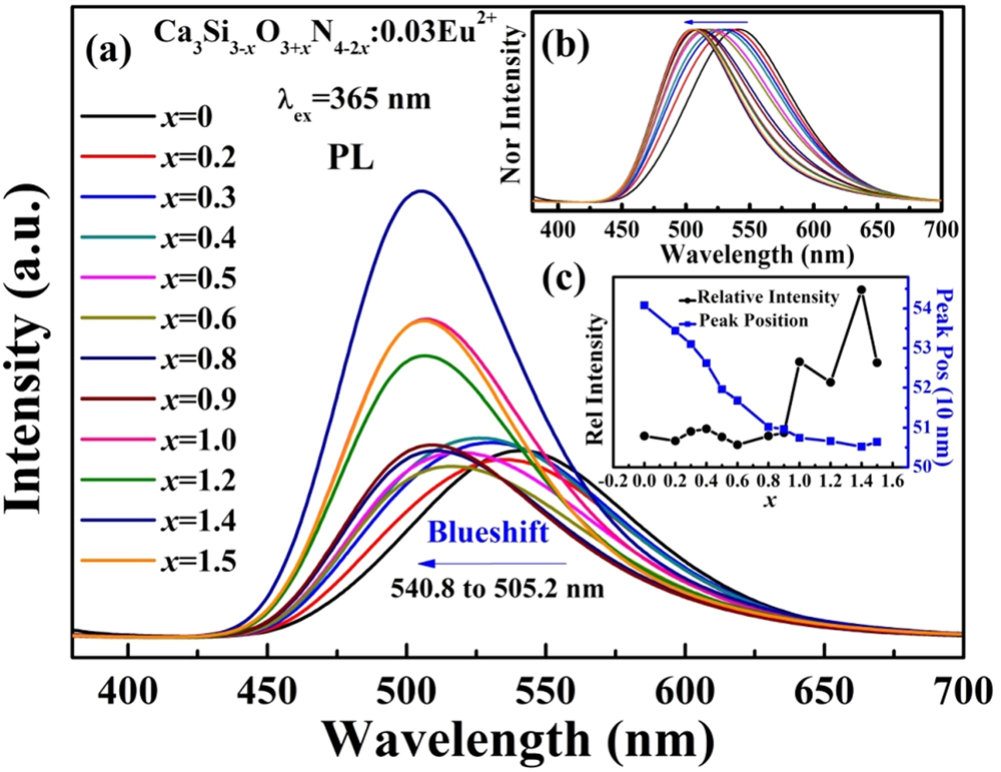
(**a**) PL and (**b**) normalized PL spectra of Ca_2.97_Si_3−*x*_O_3+*x*_N_4−2*x*_: 0.03Eu^2+^ phosphors; (**c**) the relative emission intensities and peak wavelength versus *x* values.

The photoluminescence (PL) spectra of all Ca_2.97_Si_3-*x*_O_3+*x*_N_4−2*x*_:0.03Eu^2+^ phosphors are shown in Fig. 9. A broad, asymmetric band was observed in the emission spectra, which correspond to the allowed 4f^6^5d^1^→4f^7^ electronic transitions of Eu^2+^. Figure 9(b) illustrates the normalized spectra and Fig. 9(c) show and the variation tendency of peak position and intensities against *x* values. It is worth mentioning that the peak present an obvious blue-shift from 540.8 to 505.2 nm with increasing *x* value. This blue-shift is ascribed to the variation of the crystal field environment surrounding Eu^2+^ ions. With the increase of *x*, nitrogen is gradually substituted by oxygen and Eu^2+^ ions may coordinate with less nitrogen and more oxygen. Compared with the oxygen, smaller electronegativity, bigger polarizability and higher formal charge of nitrogen contribute to the improvement of the covalency of bonds^5,10^. CaEu-N bonds have a higher covalency than CaEu-O, which leads to a bigger crystal field splitting and nephelauxetic effect^10^. Hence, the crystal field splitting is weakened by less nitrogen coordination and a blue-shift is observed. To further assess the variation of crystal field strength with *x* value, the average bond length and distortion index of the activator-anion polyhedron were calculated according to equations (3) and (4)^39^:

3 $${\rm{\Delta }}=\frac{1}{{\rm{n}}}\sum _{i=1}^{{\rm{n}}}{{[(d}_{{\rm{i}}}-{{\rm{d}}}_{{\rm{m}}}{)/d}_{{\rm{m}}}]}^{2}$$

4 $${{\rm{\sigma }}}^{2}=\frac{1}{{\rm{n}}-1}\sum _{i=1}^{{\rm{n}}}{{({\rm{\alpha }}}_{{\rm{i}}}-{{\rm{\alpha }}}_{{\rm{m}}})}^{2}$$

where *d*_*i*_ is the bond length of polyhedron, *d*_*m*_ is the average bond, α_i_ is bond angle of polyhedron and α_m_ represent the average bond length (90 for octahedral, 109.47 for tetrahedron and 120 for triangle). The average bond length and distortion index of the activator-anion polyhedron are listed in Table 2 and Table 3, respectively. We can see that the distortion index increase with decreasing *x* value except for several individual ones. The average bond length also increases with decrease in *x* value because distortion can induce an increase in average bond length of the polyhedron. According to the Jahn-Teller effect^40^, distortion of a polyhedron will lead to further crystal field splitting of center activator, as well as a red-shift of luminescent emission^41^. That may be another reason for the enhancement of crystal field splitting in Fig. 8 and the blue-shift of emission in Fig. 9. All these results are in well agreement and it’s reasonable to conclude that the crystal field strength decreases with increasing *x*.

**Table 2:** Average bond length (unit: Å) of Ca/Eu-O/N polyhedron in Ca_2.97_Si_3−*x*_O_3+*x*_N_4-2*x*_:0.03Eu^2+^ (*x* = 0, 0.5 and 1) phosphors.

Ca Sites	Ca/Eu1	Ca2	Ca3	Ca4	Ca5	Ca6	Ca7
*x* = 0	2.474	2. 310	2.283	2.551	2.678	2.730	2.676
*x* = 0.5	2.440	2.234	2.816	2.676	2.720	2.600	2.587
*x* = 1	2.077	1.925	2.577	2.535	2.390	2.473	2.488

**Table 3:** Distortion index of the Ca/Eu-O/N polyhedron in Ca_2.97_Si_3−*x*_O_3+*x*_N_4−2*x*_: 0.03Eu^2+^ (*x* = 0, 0.5 and 1) phosphors.

Sample	Ca1		Ca2		Ca3		Ca4		Ca5		Ca6	Ca7
	Δ × 10^3^	σ^2^	Δ × 10^3^	σ^2^	Δ × 10^3^	σ^2^	Δ × 10^3^	σ^2^	Δ × 10^3^	σ^2^	Δ × 10^3^	Δ × 10^3^
*x* = 0	0	242.75	18.74	585.23	7.67	987.82	3.07	107.61	22.30	448.72	57.23	18.15
*x* = 0.5	0	3.40	49.82	318.07	4.52	260.04	12.57	139.19	17.58	282.46	20.13	7.47
*x* = 1	0	1.82	35.08	184.04	0.15	100.93	24.88	807.17	14.01	266.39	4.44	5.71

**Figure 10:**
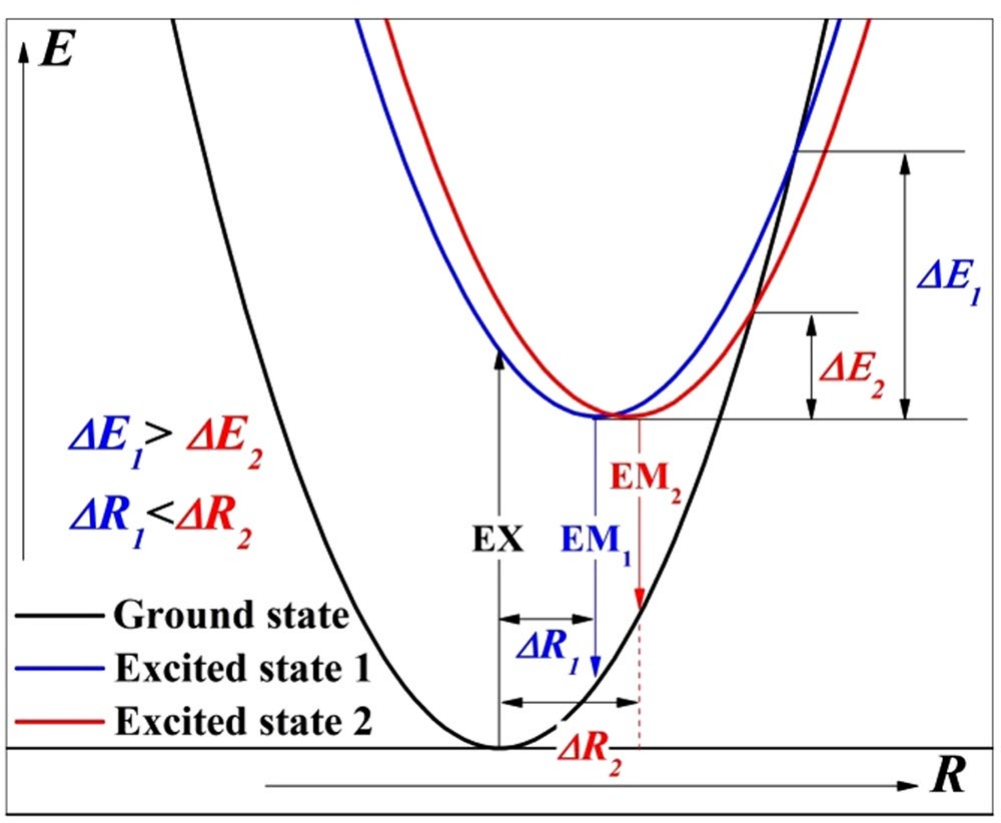
Configuration coordinate diagram showing the relationship between luminescence process and EX (excition), EM (emission), *ΔR* (shift of Configuration coordinate), *ΔE* (activation energy for thermal quenching).

To further investigate the luminous properties, the ΔSS, full width at half maximum (FWHMs) of excitation and emission spectra as well as crystal field splitting (CFS) are calculated and listed in Table 4. The ΔSS was calculated according to the energy difference between the maximal emission and excitation, and the CFS was calculated by the gap between the first and the last component peaks of the PLE spectra^42^. With the increase in *x*, the FWHMs of excitation spectrum decrease from 162 nm to 124 nm and the CFS decrease from 15133 cm^−1^ to 11219 cm^−1^. The two variation tendencies are perfectly anastomotic. As discussed above, the decrease of CFS is attributed to the weakened crystal field strength surrounding Eu^2+^ ions, which certainly lead to a decrease in FWHMs of excitation spectrum. By contrast, the FWHMs of emission spectra increase from 97 to 104 nm, then decrease to 77 nm with increase in *x*. The ΔSS shows the similar variation tendencies with that of FWHMs. The FWHMs of emission spectra are closely related to the symmetry of center sites and formation of glass phase. Lattice distortion weakens the symmetry of center sites and changes the coordination environment of center ions, which leads to a weak excursion of emission compared with the original state. Thus, the emission bands are extended. In addition, glass phase has the merit of short-range order and long-range disorder, which possess abundant and continuously distinguishable sites available for luminescent activators^43,44^. These abundant sites may also broaden the emission bands. The Stokes shift is an important feature of luminescence. The configuration coordinate diagram explaining luminescence process is shown in Fig. 10. It can be seen from Fig. 10 that a larger shift of coordinate diagram (*ΔR*) will result in a larger Stokes shift and smaller *ΔE*. The *ΔE* is the activation energy for thermal quenching. Accordingly, thermal stability of phosphors can be speculated via variation of Stokes shift. In this way, the sample *x* = 0.3 is considered to have the best thermal stability. The thermal stability of other samples shows a trend of decreasing with *x* skewing the optimum value (0.3). To obtain high luminescence, small Stokes shifts are desired to avoid strong overlap between the absorption and emission bands and to achieve small thermal quenching.

**Table 4:** Excitation and emission peaks, stokes shift (SS), and the crystal field splitting (CFS) of the Ca_2.97_Si_3−*x*_O_3+*x*_N_4−2*x*_: 0.03Eu^2+^ phosphors.

*x* value	FWHM (nm)	FWHM (nm)	ΔSS (cm^−1^)	CFS (cm^−1^)
0	162	97	11060	15133
0.2	152	102	11390	14696
0.3	147	103	11693	13901
0.4	137	104	11502	13795
0.5	130	100	11448	13192
0.6	126	99	11514	13143
0.8	120	90	11245	12884
0.9	119	88	11298	12757
1	121	82	11309	12993
1.2	119	82	11182	11637
1.4	124	76	11032	11837
1.5	124	77	10834	11219

**Figure 11:**
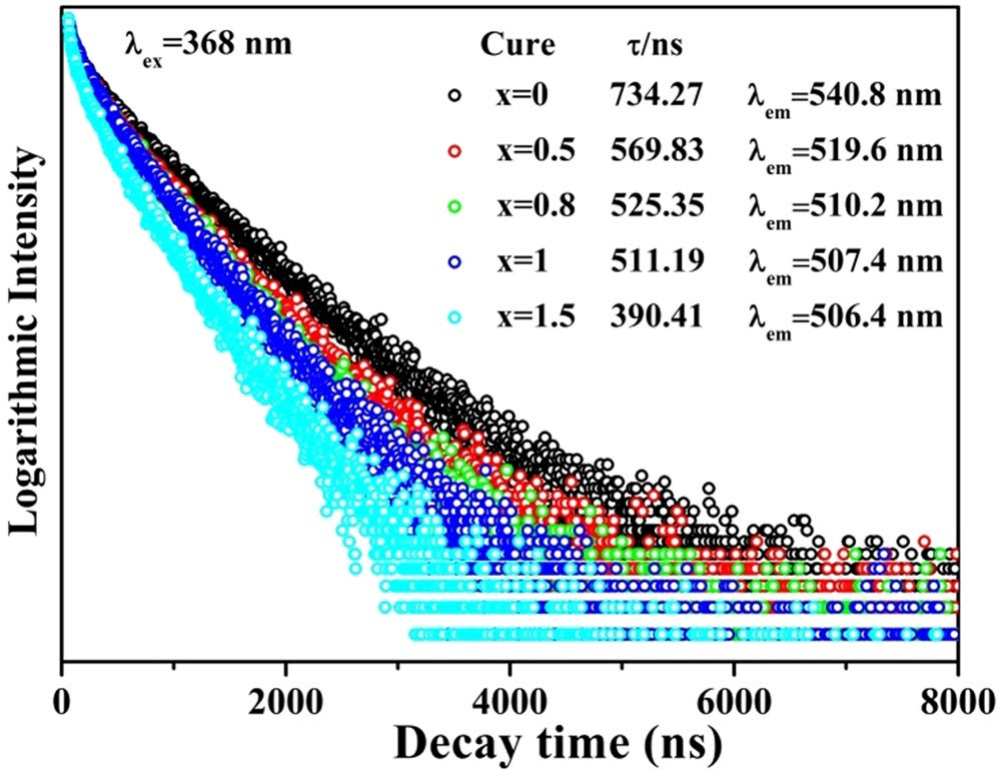
Room temperature decay curves of Ca_2.97_Si_3−*x*_O_3+*x*_N_4−2*x*_: 0.03Eu^2+^ (*x* = 0, 0.5, 0.8, 1, 1.5) phosphors.

For further investigation of the effect of structural evolution on the luminescence properties, the PL decay curves of Eu^2+^ in Ca_3_Si_3-*x*_O_3+*x*_N_4-2*x*_:0.03Eu^2+^ (*x* = 0, 0.5, 0.8, 1, 1.5) phosphors were depicted in Fig. 11. All decay curves could be well fitted via the single-exponential decay equation:

5 $${I}={{I}}_{{0}}+{Aex}\,{p}(-t/\tau )$$

where *I*_0_ represents the background intensity and τ is the lifetime. With increase in *x* values, the lifetimes of Eu^2+^ emissions were determined to be 734.27, 569.83, 525.35, 511.19 and 390.41 ns, respectively. The decay times sharply reduce with increase in *x* value, which indicates the increasing possibility of non-radiative transition caused by energy transfer among the Eu^2+^. It has been reported that the energy of an excited Eu^2+^ ion is likely to be consumed by another neighboring Eu^2+^ or the traps caused by the defects if the distances between these are close enough via a non-radiative way, resulting in a lifetime reduction^43,45^. With the crystallite enlargement, more and more activator ions are located in the interior of the crystals, rather than at the near surface where energy can rapidly transfer to surface defects and then be consumed by high vibrational energies^34^.

**Figure 12:**
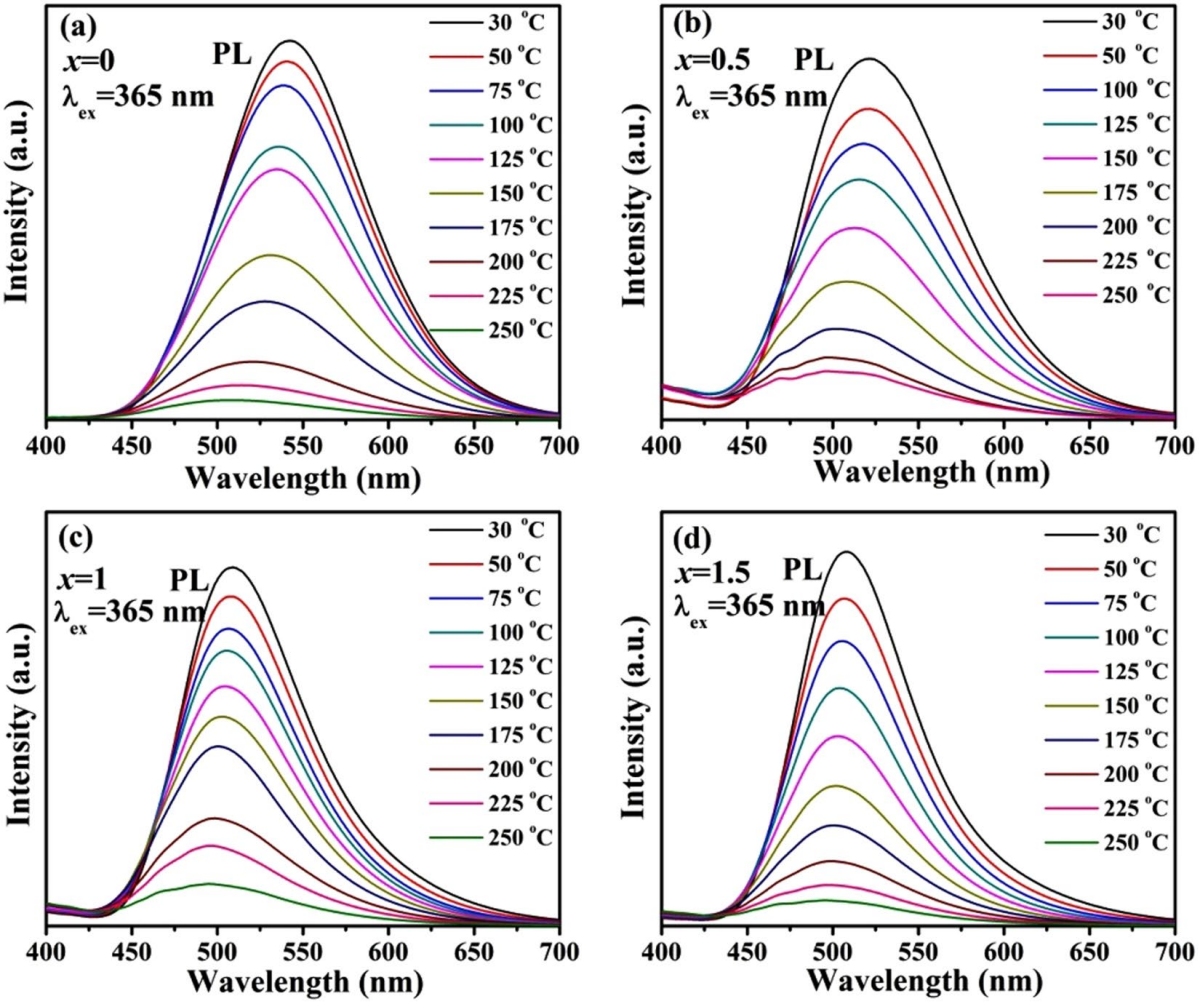
(**a**–**d**) Temperature dependence of emission spectra of Ca_2.97_Si_3−*x*_O_3+*x*_N_4−2*x*_:0.03Eu^2+^ (*x* = 0, 0.5, 1 and 1.5, respectively) phosphors.

**Figure 13:**
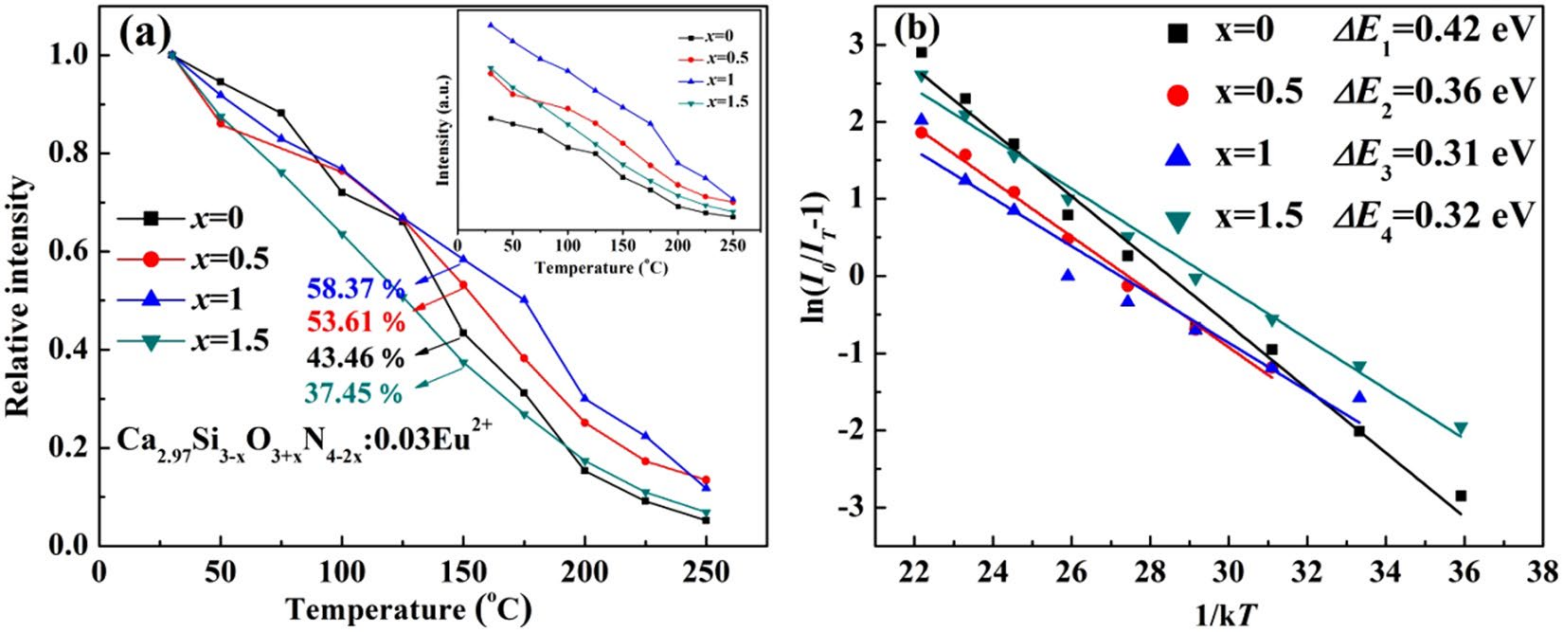
(**a**) The dependence of the emission intensities on temperatures; (**b**) The fitting plot of ln (I_0_/I_T_ − 1) and 1/kT of Ca_2.97_Si_3-*x*_O_3+*x*_N_4-2*x*_: 0.03Eu^2+^ (*x* = 0, 0.5, 1 and 1.5, respectively) phosphors.

Thermal quenching is one of the most important factors determining the utility of a phosphor. Based on this, the temperature dependence of emission spectra of four selected samples Ca_2.97_Si_3−*x*_O_3+*x*_N_4−2*x*_: 0.03Eu^2+^ (*x* = 0, 0.5, 1 and 1.5) is illustrated in Fig. 12. To further estimate the thermal stability of the four samples, the dependence of the emission intensities on temperatures and the activation energy (*ΔE*) for thermal quenching are shown in Fig. 13. The *ΔE* was calculated according to Arrhenius equation^46^:

6 $${{\rm{I}}}_{{\rm{T}}}=\frac{{{I}}_{{0}}}{1+{c}\,{\exp }(-\frac{{\Delta }E}{{kT}})}$$

where *I*_0_ and *I*_*T*_ are the emission intensity of the phosphor at room temperature and measurement temperature *T*, respectively; c is the ratio of attempt rate (typically 3 × 10^13^ Hz as the maximum phonon frequency) and radiative decay rate of the 5 d state of Eu^2+^ (typically 1.1 × 10^6^ Hz), and k is the Boltzmann constant (8.617 × 10^−5^ eV K^−1^). One can see from Fig. 13 that when the temperature reaches 150 °C, the luminous intensities remain 58.37%, 53.61%, 43.46% and 37.45% of that at room temperature, respectively. The *ΔE* of homologous samples were calculated to be 0.31, 0.36, 0.42 and 0.32 eV, respectively. It’s clear that thermal quenching is weakened when *x* values deviate from the optimal one (*x* = 1). Generally, the rigid three-dimensional structure is believed to be the main reason for small thermal quenching of (oxy)nitrides, though the exact mechanism is still not exhaustive. To quantify structural rigidity of title phosphors, Debye temperatures and Young’s modulus of the four samples Ca_2.97_Si_3-*x*_O_3+*x*_N_4-2*x*_: 0.03Eu^2+^ (*x* = 0, 0.5, 1 and 1.5) were calculated by first principle method. The corresponding results are listed in Table 5. It can be seen that the both the Debye temperatures and Young’s modulus decrease when *x* deviates from 1, indicating that the sample *x* = 1 possesses the best structural rigidity. The structural rigidity of the phosphors weakens when *x* deviates from 1. Debye temperature (Θ_D_) and average atomic displacement (U_iso_) have the relationship as described in equation (7)^47^:

7 $${{\rm{\Theta }}}_{D,{\rm{i}}}=\sqrt{\frac{{{\rm{3h}}}^{2}{{\rm{TN}}}_{{\rm{A}}}}{{{\rm{A}}}_{{\rm{i}}}{{\rm{k}}}_{{\rm{B}}}{{\rm{U}}}_{\text{iso},{\rm{i}}}}}$$

It’s found that a large U_iso_ means a low Θ_D_ and poor structural rigidity. The variation of Debye temperature and Young’s modulus is in agreement with that of the average bond length and lattice distortion shown in Tables 2 and 3, respectively. Based on that, it’s reasonable to conclude that the weakened thermal stability with deflected *x* values may be attributed to the lattice distortion, which has been clarified in Fig. 3. The lattice distortion damaged the rigid three-dimensional structure of the host, which leads to a bigger thermal quenching.

**Table 5:** Debye temperatures and Young’s modulus of Ca_2.97_Si_3−*x*_O_3+*x*_N_4−2*x*_: 0.03Eu^2+^ (*x* = 0, 0.5, 1 and 1.5) phosphors.

Sample	*x* = 0	*x* = 0.5	*x* = 1	*x* = 1.5
Debye temperature (K)	760.12	787.18	796.35	734.24
Young’s modulus	238.55	251.90	258.73	225.69

**Figure 14:**
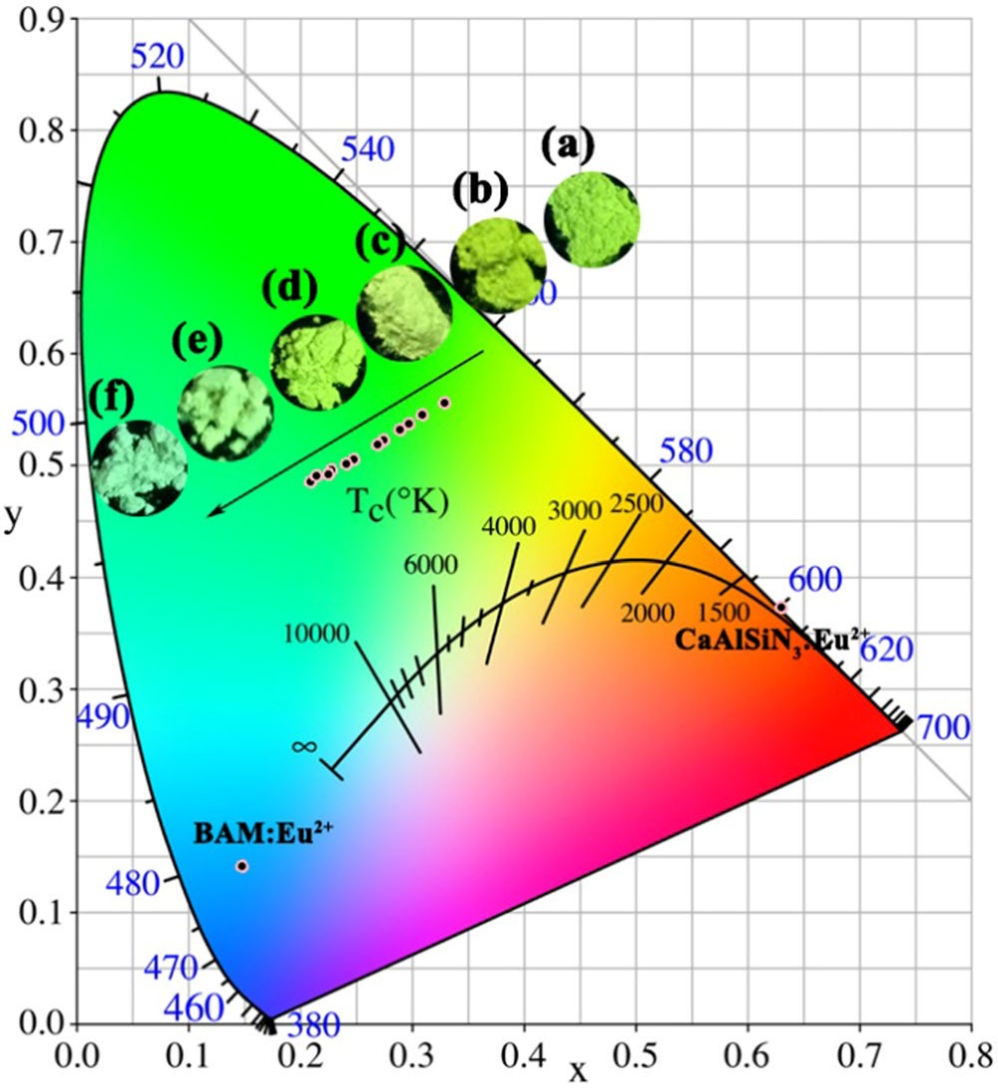
CIE chromaticity diagram of all Ca_3_Si_2_O_4_N_2_:0.08Ce^3+^, *x*Tb^3+^ phosphors; the inset (**a**)~(**f**) show the digital images corresponding different samples (*x* = 0, 0.3, 0.5, 0.8, 1.2, 1.5), respectively; CIE chromaticity diagram of commercial blue BAM: Eu^2+^ and red CaSiAlN_3_: Eu^2+^ phosphors are also given.

To show the real color of as-prepared phosphors, color rendering index (CIE) chromaticity coordinates of as-prepared Ca_2.97_Si_3-*x*_O_3+*x*_N_4-2*x*_: 0.03Eu^2+^ phosphors, commercial blue BAM: Eu^2+^ and red CaSiAlN_3_: Eu^2+^ phosphors are calculated and given in Fig. 14. The insets show digital images of different samples under 365 nm excitation. It can be seen that the CIE coordinates move from yellow-green (0.3292, 0.5556) to blue-green (0.2149, 0.4905) with increase in *x* values due to a blue-shift of emission. The shift of color points demonstrates that wavelength-tunable green phosphors can be produced by controlling the crystal structure of the given host.

To estimate the practicability of title phosphors, the quantum efficiency (QE) of selected samples (*x* = 0, 0.2, 0.3, 0.4, 0.8, 1, 1.2, 1.4 and 1.5) is measured to be 5.14%, 12.22%, 7.60%, 11.65%, 19.17%, 19.16%, 34.60%, 13.82% and 5.12%, respectively. It’s noted that the QEs of samples tend to be larger when *x* come close to 1 (the original composition Ca_3_Si_2_O_4_N_2_), while the QEs tend to be smaller when *x* diverge from 1. This may be caused by the lattice distortion. When *x* diverges from 1, change of solid solubility (*x* value) the distortion of crystal lattice may produce some defects, which accelerates quenching of luminescence via energy transfer^48^. Higher QEs of title phosphor can be obtained by improving synthesis condition.

**Figure 15:**
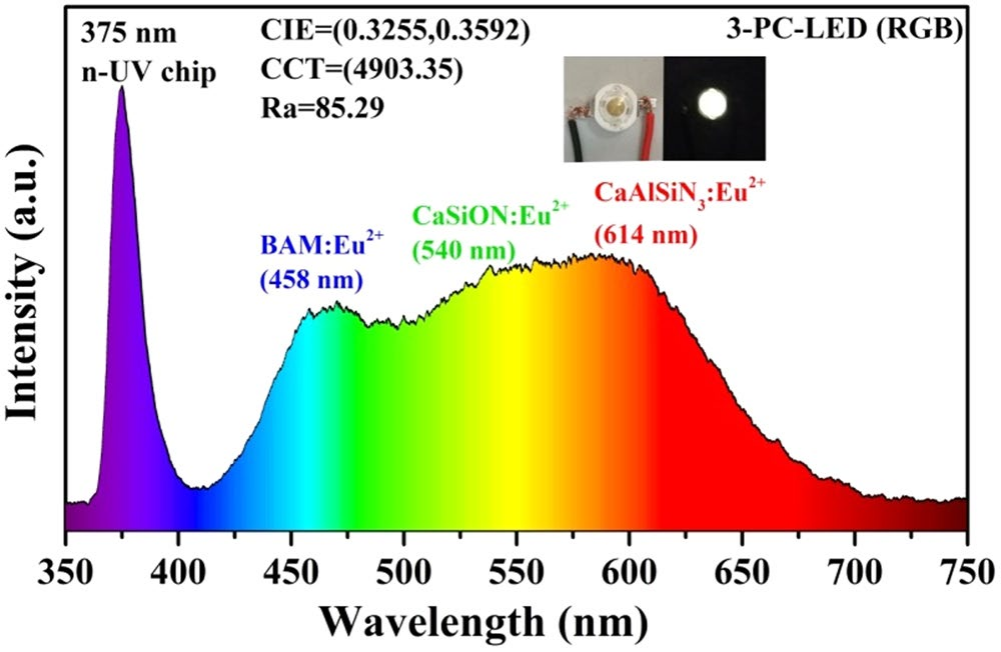
Electroluminescent spectrum of self-made w-LED white fabricated by a n-UV chip (375 nm), together with Ca_3_Si_3_O_3_N_4_: 0.03Eu^2+^, commercial blue BAM: Eu^2+^ and red CaAlSiN_3_: Eu^2+^ phosphors driven by 30 mA current.

To further demonstrate the potential application of title phosphors, the self-made lamp was fabricated by coating green Ca_3_Si_3_O_3_N_4_: 0.03Eu^2+^ (*x* = 0) and commercial blue BAM: Eu^2+^ and red CaAlSiN_3_: Eu^2+^ phosphors on a n-UV chip (λ_ex_ = 375 nm). The electroluminescent spectrum of the w-LED lamp was displayed in Fig. 15. It can be seen that the electroluminescent spectrum covers full visible light with blue, green and red region. The CIE color coordinates, CCT and Ra of the self-fabricated w-LED lamp were calculated to be (0.33, 0.36), 4903 K and 85.3, respectively. The relatively high Ra and appropriate CCT value demonstrate that the Ca_3_Si_3_O_3_N_4_: 0.03Eu^2+^ can be a promising green-emitting phosphor for application in w-LEDs.

## Conclusions

In brief, this study mainly clarifies the relationship between crystal structure variation and luminescence properties of (oxy)nitride Ca_3_Si_3−*x*_O_3+*x*_N_4−2*x*_: Eu^2+^ phosphors. When the *x* value diverges the optimum one (*x* = 1), lattice is gradually distorted and symmetry of local environment decreased. This lattice distortion is conducive to generating a broader emission spectrum, but leads to an increase in ΔSS, causing a bigger thermal quenching. The particle size decreases with increase in *x* values, which is considered to be the reason for the sharp decline of lifetimes. Enhanced crystal field splitting leads to an obvious red-shift and a broader excitation spectrum, making it possible to serve as green phosphor for n-UV LEDs. The fabricated w-LED lamp exhibits high CRI (Ra = 85.29) and suitable CCT (4903.35 K). These results indicate that green phosphor for n-UV w-LEDs with tunable spectrum can be obtained by the provided strategy, via controlling the crystal structure and morphology.
